# Supplementary material for: Widespread chromatin context-dependencies of DNA double-strand break repair proteins
Source: Nat Commun. 2024 Jun 22;15:5334. doi: 10.1038/s41467-024-49232-x (PMC11193718; doi:10.1038/s41467-024-49232-x)
Supplement: Supplementary file 1 — Supplementary Information [file 41467_2024_49232_MOESM1_ESM.pdf]

SUPPLEMENTARY METHODS **2**
SCREEN PROCEDURE 2
Liquid handling *2*
*Day 1: Induction of Cas9 and transfection of KO gRNA library (**scheme 1**)* *2*
*Day 5: passaging of cells and quality checks (**scheme 2**)* *3*
*Day 6: induction of DSBs in IPRs by transfection with LBR2 gRNA (**scheme 3**)* *4*
*Day 9: cell lysis and quality controls (**scheme 4**)* *4*
Screening replicates *5*
*Downstream processing: sample preparation for IPR sequencing (**scheme 5**)* *5*
PROCESSING AND STATISTICAL ANALYSIS OF K562 SCREEN DATA. 7
1. Demultiplexing and general quality control of sequencing reads. *8*
2. Scoring of indels in IPRs. *8*
3. Calculation of changes in MMEJ:NHEJ balance *8*
4. Identification of proteins with global effects on MMEJ:NHEJ balance. *9*
5. Identification of proteins with CCD: three-step linear modelling *10*
6. Estimation of chromatin context dependent MMEJ:NHEJ balance changes. *12*
7. Estimation of screen KO penetrance *12*
8. Data visualization *13*
SUPPLEMENTARY TABLES **14**
SUPPLEMENTARY TABLE 1: GRNA AND PRIMER SEQUENCES USED IN THIS MANUSCRIPT. 14
SUPPLEMENTARY TABLE 2: OVERVIEW OF SEQUENCING READ NUMBERS PER SAMPLE IN THE SCREEN 14
SUPPLEMENTARY FIGURES **15**
SUPPLEMENTARY FIGURE 1: SCREEN REPLICATE REPRODUCIBILITY AND DISTRIBUTION OF ΔLOG$_2$MMEJ:NHEJ VALUES. 15
SUPPLEMENTARY FIGURE 2: Z-TRANSFORMATION AND COMBINING OF REPLICATE MEASUREMENTS OF LOG$_2$MMEJ:NHEJ VALUES. 16
SUPPLEMENTARY FIGURE 3: PRINCIPAL COMPONENT REGRESSION ANALYSIS. 18
SUPPLEMENTARY FIGURE 4: EXAMPLES OF LINEAR FIT CORRELATION WITH INDIVIDUAL CHROMATIN FEATURES. 19
SUPPLEMENTARY FIGURE 5: CCD PATTERN OF DNA REPAIR OF THE SAME GENE ONTOLOGY CATEGORY 21
SUPPLEMENTARY FIGURE 6: ESTIMATION OF GENOME-WIDE DYNAMIC RANGES OF CHROMATIN FEATURES. 22
SUPPLEMENTARY FIGURE 7: EFFECT SIZE AND KNOCK-OUT PENETRANCE ESTIMATION. 23
SUPPLEMENTARY FIGURE 8: M- AND N-SYNERGIES IN RPE-1 CELLS. 25
SUPPLEMENTARY FIGURE 9: ATM AND DNAPK INHIBITOR EFFECTS. 26
SUPPLEMENTARY FIGURE 10: IMPACT OF CCDS ON HUMAN TUMOR GENOMES. 27
SUPPLEMENTARY REFERENCES **28**

SUPPLEMENTARY METHODS

Screen procedure

Liquid handling

Steps in the procedure were performed in a semi-automated fashion either with MicroLab STAR liquid handler (Hamilton Company, **blue in scheme 1-5**), Multidrop™ Combi Reagent Dispenser (ThermoFisher, **green in scheme 1-5**) or manually (**grey in scheme 1-5**).

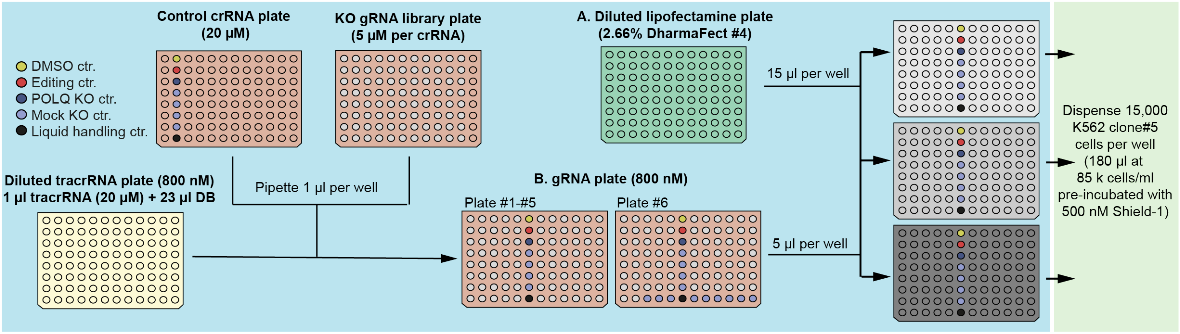

Scheme 1: Day 1 procedure – KO gRNA library transfection (repeated 6 times). 24 µl of 800 nM tracrRNA (yellow) was pipetted to the final crRNA:tracrRNA plate (orange). Then, 1 µl of KO gRNA library or control plate added to screening plate position. Next, Diluted DharmaFect #4 was pipetted to lipofectamine plate (green). 15 µl of the lipofectamine was pipetted into three 96-well plates (three replicates) and 5 µl of the crRNA:tracrRNA mix added later. With the cell dispenser, 15,000 clone5 cells per well were dispensed (green).

*Day 1: Induction of Cas9 and transfection of KO gRNA library (**scheme 1**)*

Eight hours before the KO gRNA library transfection, we diluted clone 5 to a final concentration of 85,000 cells/ml with medium containing 500 nM Shield-1 (Aobious cat. no. AOB1848) to stabilize DD-Cas9 protein. As first step in the KO gRNA library transfection, we diluted 20 µM tracrRNA (IDT cat. no. 1072534) stock concentration to 800 nM in Duplex Buffer (DB, IDT cat. no. 11-01-03-01) in a final volume of 24 µl. Next, we pipetted 1µl crRNA of KO gRNA library (stock at 20 µM in DB) or controls to its appropriate position in the gRNA plate (orange, scheme 1). Plates 1 to 5 in the screen included 88 KO gRNAs and 8 controls wells: four mock KO controls (crRNA was omitted), one POLQ KO gRNA control (used as a positive control, sequences in **Supplementary Table 1**), one editing control (transfected with LBR2 gRNA [1]) and one pipetting control. In the pipetting control, 1 µl phenylarsine oxide (PAO Sigma-Aldrich, cat. no. P3075, stock concentration of 10 mM) was pipetted instead of 1 µl crRNA. 10 µM of PAO is enough to kill K562 cells, so we used visual inspection of cell death at day 5 to check if the KO gRNA library pipetting step was successful. Plate 6 included nine additional mock KO controls, making a total of 33 per replicate. In parallel, we diluted DharmaFect #4 (Horizon Discovery, cat. no. T-2004-03) lipofectamine to 2.66% (0.4 µl in 15 µl) with Optimem (Gibco, cat. no. 31985070). After 5 minutes incubation at room temperature, 15 µl of diluted DharmaFect #4 was pipetted in the three empty 96-well V-bottom plates (Thermo Fisher, cat. no. 11816003) and 5 µl of the 800 nM coupled gRNA. This mix was incubated for 15 minutes at room temperature and subsequently 15,000 clone 5 cells were dispensed per well (180 µl

of clone 5 cells in 500 nM Shield-1). This procedure was repeated six times, once for each different KO gRNA library plate. We note that every new batch of DharmaFect #4 lipofectamine was tested and the cell:lipofectamine ratio was adapted for optimal transfection efficiencies. The reagent quantities described above are representative of the concentrations used in the screen.

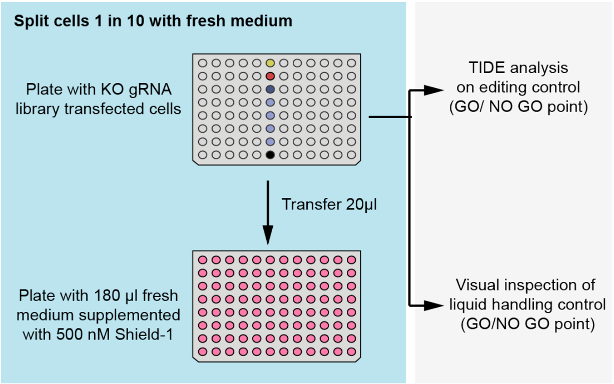

Scheme 2: Day 5 procedure - Passaging of cells and harvesting of control samples (repeated 18 times). Screening plates were split 1:10 with fresh medium. Editing control was harvested for TIDE analysis and the pipetting control was checked visually.

*Day 5: passaging of cells and quality checks (**scheme 2**)*

Four days after KO gRNA library transfection, we split transfected cells 1:10 with fresh medium supplemented with 500 nM Shield-1. At this step, we harvested editing control wells for TIDE analysis and we visually inspected cell death in the pipetting control wells. We used these two quality controls to assess if specific plates should be discarded or kept for the following steps. We repeated this process for every plate in the screening.

TIDE [2] was used to monitor the editing efficiency prior to high-throughput sequencing, as follows. Editing control wells were harvested and cells were lysed with 30 µl DirectPCR lysis buffer (Viagen cat. no. 301-C) supplemented with 1 mg/ml proteinase K (Bioline, cat. no. BIO-37084) by incubating them at 55 °C for at least 2 hours up to overnight, followed by heat inactivation for 45 min at 85 °C. To monitor the CRISPR editing frequency, we used primers spanning the endogenous LBR2 target site as previously reported [1]. PCR was performed using 10 µl MyTaq Red mix (Bioline, cat. no. BIO-25044), 1 µM of each TAC0017 and TAC0018 primers, 2 µl of cell lysate and up to 20 µl of water. PCR conditions for TIDE analysis are the following ones: 1 min at 95 °C followed by 28 cycles of 15 s at 95 °C, 15 s at 58 °C and 30 s at 72 °C and a final extension of 1min at 72 °C. The excess of PCR primers was degraded by EXOSAP treatment as follows. For each 10 µl of PCR reaction, 0.125 µl of Shrimp Alkaline Phosphatase (1 U/ml; New England Biolabs, cat. no. M0371S), 0.0125 µl Exonuclease I (20 U/ml; New England Biolabs, cat. no. M0293S) and 2.36 µl of water were added. Samples were incubated at 37 °C for 30 min and heat inactivated for 10 min at 95 °C. Next, 5 µl of EXOSAP-treated PCR mix was Sanger sequenced with 5 µl of TAC0017 primer

at 5 µM concentration by Macrogen (EZ-seq). The resulting Sanger sequence traces were analyzed using the TIDE algorithm [2] to determine the editing efficiency.

*Day 6: induction of DSBs in IPRs by transfection with LBR2 gRNA (**scheme 3**)*

We manually mixed LBR2 crRNA (crRNA targeting DSB-TRIP reporters) with tracrRNA at a final concentration of 800 nM in DB and diluted DharmaFect in Optimem (2.66% concentration). Then, we pipetted 15 µl of diluted lipofectamine into six empty 96-well plates and added 5 µl of LBR2 crRNA:tracrRNA. After incubating this mix for 15 min at room temperature, we added 180 µl KO cells in arrayed format. We repeated this procedure for each replicate independently with freshly prepared LBR2 gRNA and DharmaFect #4 mixes.

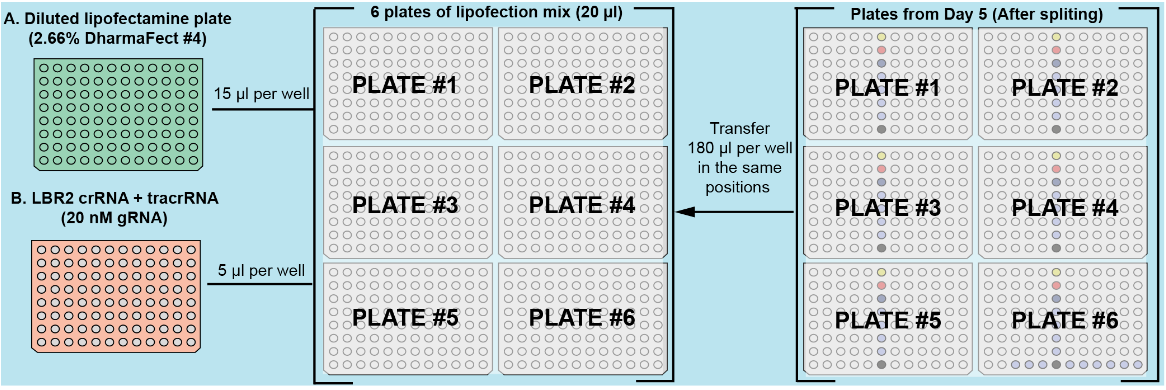

Scheme 3: Day 6 procedure - LBR2 gRNA transfection (repeated 3 times). DharmaFect #4 and LBR2 crRNA:tracrRNA were manually diluted. Then, 15 µl DharmaFect #4 and 5 µl LBR2 crRNA:tracrRNA (B on A) was combined into each well of 6 empty 96-well plates. 15 minutes after mixing, 180 µl of cells, split on day 5, were transferred into the transfection mix plate.

*Day 9: cell lysis and quality controls (**scheme 4**)*

Three days after LBR2 gRNA transfection we harvested the screening plates. To do so, we centrifuged 96-well plates to pellet the cells (300 g for 5 min). Then, we removed the supernatant and pipetted 30 µl of DirectPCR lysis buffer supplemented with 1 mg/ml proteinase K on the cell pellets. After a couple of pipetting cycles to mix cells with lysis buffer, we transferred the cell lysate to an empty 96-well PCR plate (ThermoFisher, cat. no. AB0900). Cells were lysed overnight at 55°C in a thermocycler, and proteinase K was subsequently inactivated for 45 minutes at 85°C.

To monitor CRISPR editing efficiency of the second transfection, we performed TIDE analysis with cell lysate from a random well from each plate, as described above (Day 5).

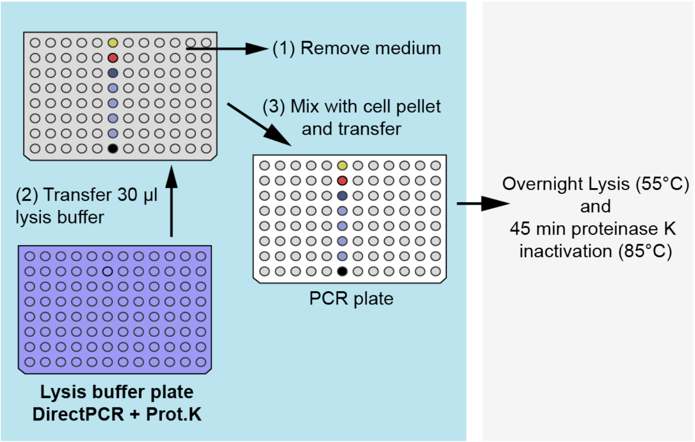

Scheme 4: Day 9 procedure - Screening plate harvesting (repeated 18 times). Harvesting was performed in three steps. After cells were pelleted by centrifugation, 170 µl supernatant was removed (grey, 1); lysis buffer was added (purple) and mixed with cell pellets (grey, 2); and the cell lysate was transferred to empty PCR plates (white, 3). Cells were lysed overnight at 55°C and proteinase K was subsequently heat inactivated for 45 min at 85 °C.

Screening replicates

The screening was performed twice, more than a month apart. Each time the screening was performed in three replicates with independent transfection mixes. Three out of six replicates were discarded because of technical reasons such as wrong liquid handling, unsuccessful transfection (at least ~50% editing in the editing efficiency control) or problems during sample processing. One replicate from the first screen and two replicates from the second screen passed the quality controls. We refer to these replicates as replicate 1 (R1), replicate 2 (R2) and replicate 3 (R3).

*Downstream processing: sample preparation for IPR sequencing (**scheme 5**)*

For the sequencing of the IPRs (to identify indels and their linked IPR barcodes) in all screen samples, we employed a two-step PCR indexing and pooling strategy as previously described [3], with some adaptations. We performed the first PCR reaction (indelPCR1) with TAC0007 (indexed) and TAC0012 (non-indexed) primers with a unique TAC0007 indexed primer for each 96-well plate, and the second PCR reaction (indelPCR2) with TAC009 (non-indexed) and TAC0159 (indexed) primers with 96 different TAC0159 primers (one for each well in a 96-well plate). Pipetting was performed using the MicroLab STAR liquid handler (Hamilton Company).

IndelPCR1 and indelPCR2 were performed under similar PCR conditions with the only difference being the number of cycles. In both reactions, a denaturing step was performed for 1 min at 95 °C, low annealing temperature amplification cycles (cold cycles) for 15 s at 95 °C, 15 s at 55 °C and 15 s at 72 °C, high annealing temperature amplification cycles (hot cycles) for 15 s at 95 °C, 15 s at 70 °C and 15 s at 72 °C and a final extension of 2 min at 72 °C.

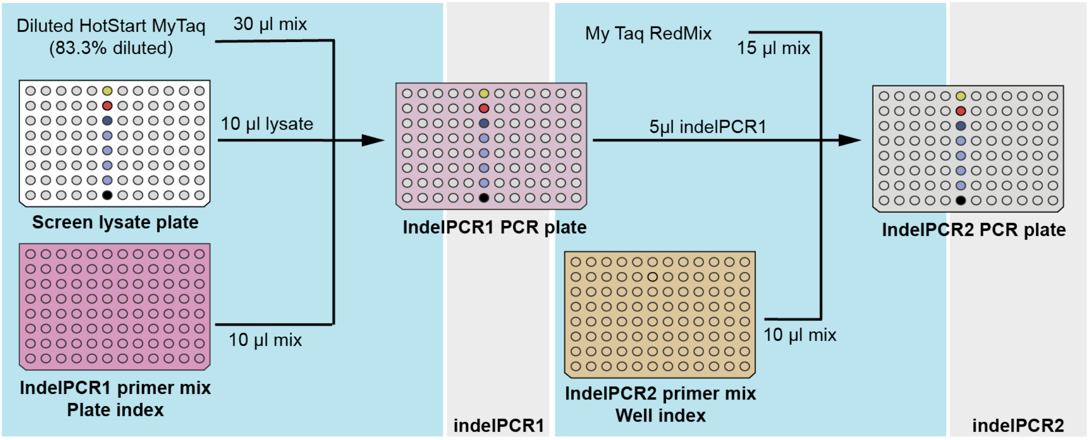

Scheme 5: Screening sample preparation for sequencing. PCR amplification of indel and barcode of each IPR in all screening samples was performed in two steps: indelPCR1 and indelPCR2. Pipetting was performed with the liquid handler (blue) and PCRs with a ThermoCycler (grey). IndelPCR1 was pipetted from three source plates: Diluted HotStart MyTaq Red Mix (5 parts of mix with 1 part of H2O), screening cell lysates (white) and indelPCR1 primer mix (purple). A different indexed primer was used per plate. IndelPCR2 was also pipetted from three source plates: MyTaq Red mix, indelPCR1 PCR plate (light purple) and indelPCR2 primer mix (gold). A different indexed primer was used for each well.

We performed indelPCR1 with 10 µl of cell lysate from the screening plates, 30 µl of 86.6% (5:1) diluted MyTaq HotStart Red Mix in water (Bioline, cat. no. BIO-25048) and 10 µl of 1 µM of each primer (TAC0007 and TAC0012 final concentration of 200 nM) for 4 cold cycles and 9 hot cycles. Then, we performed indelPCR2 with 5 µl of indelPCR1 product, 15 µl of MyTaq Red mix (Bioline, cat. no. BIO-25044) and 10 µl of 500 nM of each primer (TAC0009 and TAC0159, final concentration of 166 nM) for 3 cold cycles and 8 hot cycles.

Next, we pooled indelPCR2 products per plate in equal volumes and DNA was purified with cleanPCR (CleanNA cat. no. CPCR-0050) beads at a 0.8:1 beads:sample ratio. Ten µl of each pool was run on a 2% agarose gel for visual inspection, and DNA concentration was quantified by Qubit DNA dsHS Assay Kit (Invitrogen, cat. no. Q32851). Equimolar concentrations of DNA per plate were pooled and the resulting product run on a 2% agarose gel. The PCR amplicon band was cut from the gel and isolated by PCR Isolate II PCR and Gel Kit (Bioline, cat. no. BIO-52060) and lastly bead-purified. Resulting preparations were sequenced on a NextSeq 550 with single-ended 150 bp reads with ~25 % of PhiX spike-in.

Processing and statistical analysis of K562 screen data.

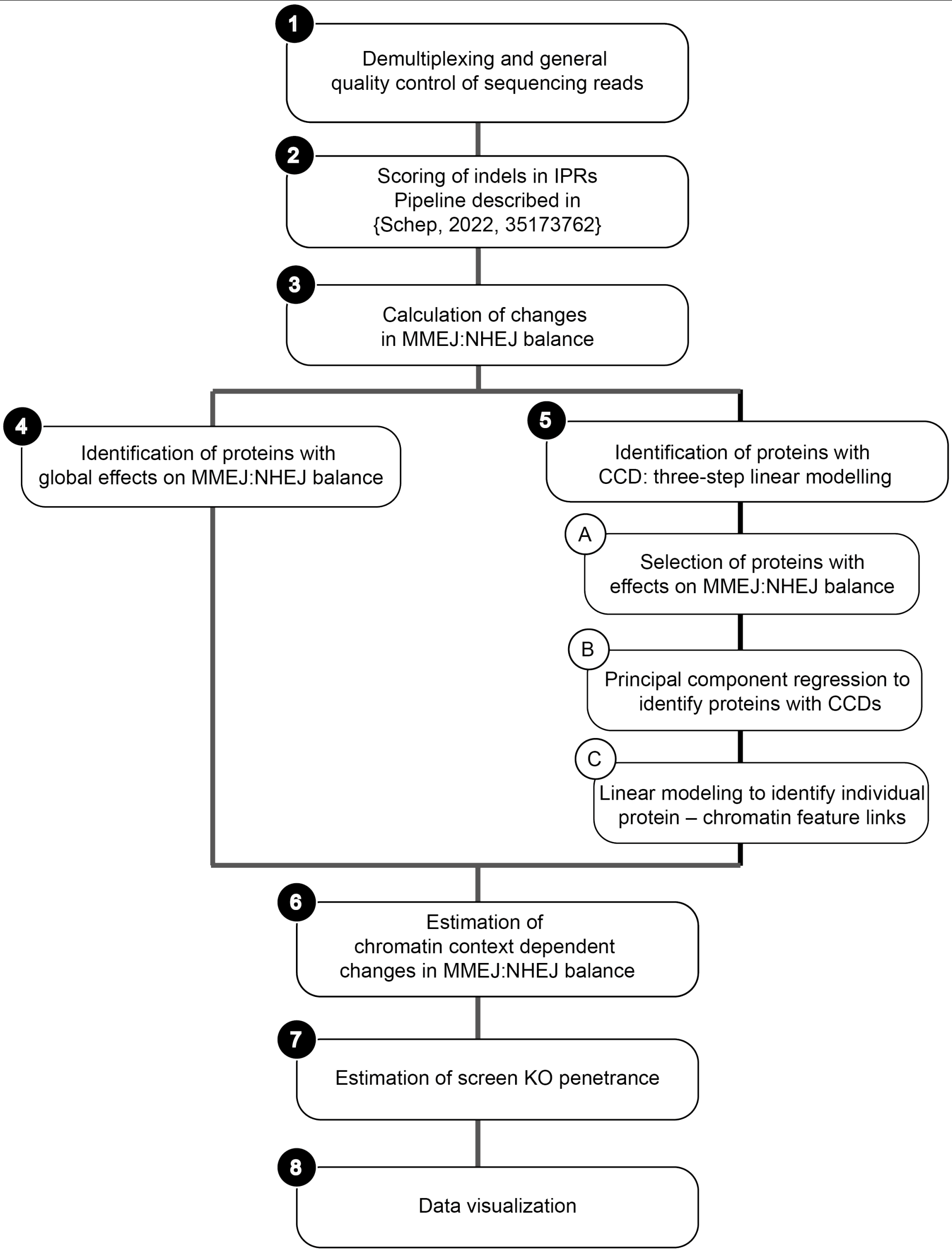

Scheme 6: Key steps in the data processing and computational analysis workflow.

1. Demultiplexing and general quality control of sequencing reads.

Demultiplexing of the sequencing reads was done based on indices added in the IndelPCR1 (plate index) and IndelPCR2 steps (well index) and each file contains the reads from a single well in the screen. We refer to these as sample throughout this section. Demultiplexed sequencing data is available in the Sequence Read Archive (https://www.ncbi.nlm.nih.gov/sra; BioProject no. PRJNA882344). An overview of obtained read numbers is provided in Supplementary Table 2.

2. Scoring of indels in IPRs.

Scoring of indels and linking to their IPR barcodes in the sequence reads was done using a previously reported computational pipeline [4]. In short, for each sequence the barcode was extracted and the indel state was classified. As documented previously [1,3], a single-nucleotide insertion was assumed to be created by NHEJ repair ($NHEJ_{ins}$), a seven-nucleotide deletion was assumed to be created by MMEJ repair ($MMEJ_{del}$), and the absence of indel was assumed to be intact DNA (uncut or perfectly repaired). In the downstream analysis, we use the number of $NHEJ_{ins}$ reads, $MMEJ_{del}$ reads and intact reads. The median and 95%-CI number of reads per replicate are summarised in Supplementary Table 2.

3. Calculation of changes in MMEJ:NHEJ balance

After indel scoring, we calculated the editing efficiency (Supplementary Equation 1) and $\log_2$ MMEJ:NHEJ balance (Supplementary Equation 2) for each individual IPR in every sample. Next, we filtered out data based on two parameters: low read numbers and low editing frequency. First, we discarded IPRs which had less than 30 reads with either $NHEJ_{ins}$ or $MMEJ_{del}$ per sample. Second, we discarded samples with an average editing efficiency lower than 25% per sample. After these filtering steps, 531 samples from R1, 541 samples from R2 and 555 samples from R3 were retained, with an average of 2.92 replicates and 18.98 IPRs per well.

$$f_{\text{Editing}} = 1 - f_{Intact} \tag{1}$$

Where f is the relative frequency, and
$intact$ is the number of reads without any indel.

$$\log_2 \text{MMEJ:NHEJ} = \log_2 \frac{\text{MMEJ}_{del}}{\text{NHEJ}_{ins}} \tag{2}$$

Where MMEJ_{del} is the number of 7 nt deletion reads, and
NHEJ_{ins} is the number of 1 nt insertion reads.

Then, we divided samples into three categories depending on the gRNA they received in the first transfection (Day 1): mock KO controls, POLQ KO controls and KO gRNA library

samples. We checked the reproducibility of the $\log_2$MMEJ:NHEJ balance between replicates (**Supplementary Figure 1A-C**). Next, we computed for each IPR the $\log_2$ fold change in MMEJ:NHEJ balance ($\Delta\log_2$MMEJ:NHEJ) as a consequence of each KO (Supplementary Equation 3) and averaged three replicates.

A negative $\Delta\log_2$MMEJ:NHEJ score implies either reduced MMEJ or increased NHEJ activity (at the tested IPR) due to the KO of the tested protein. Our assay cannot discriminate between these two possibilities, as we cannot measure the individual pathway activities – only the balance [1,3]. Likewise, a positive $\Delta\log_2$MMEJ:NHEJ score implies either increased MMEJ or decreased NHEJ activity (at the tested IPR). For simplicity, we refer to a negative $\Delta\log_2$MMEJ:NHEJ score as: the tested protein (when present) *favors MMEJ*; and we refer to a positive $\Delta\log_2$MMEJ:NHEJ score as: the tested protein (when present) *favors NHEJ*.

The POLQ KO samples provide an indication of the dynamic range of $\Delta\log_2$MMEJ:NHEJ scores that may be expected (**Supplementary Figure 1D**), because POLQ is essential for MMEJ [5,6]. On average, POLQ KO samples showed a $\Delta\log_2$MMEJ:NHEJ score of -1.58 $\log_2$ units across all IPRs, i.e., a ~3.0-fold reduction in MMEJ:NHEJ balance. As most proteins are not absolutely essential for either MMEJ or NHEJ, the dynamic range of the $\Delta\log_2$MMEJ:NHEJ scores may be expected to be less than the score observed for POLQ. Indeed, this is the case (**Supplementary Figure 1D**).

$$\Delta \log_2 \text{MMEJ:NHEJ} = \log_2 \text{MMEJ:NHEJ} - \overline{X}_{\text{MMEJ:NHEJ}} \qquad (3)$$

Where $\overline{X}_{\text{MMEJ:NHEJ}}$ is the mean $\log_2$ MMEJ:NHEJ of mock KO samples (n = 33).

We used the $\Delta\log_2$MMEJ:NHEJ scores throughout this work as a metric of the contribution of each protein to the MMEJ:NHEJ balance. For global MMEJ:NHEJ contribution of proteins, we computed the average $\Delta\log_2$MMEJ:NHEJ over all 19 pathway reporters (**Section 4 in data analysis workflow**). When calculating chromatin context dependencies (CCDs), the $\Delta\log_2$MMEJ:NHEJ of each IPR-KO combination was used (**Section 5 in data analysis workflow**).

4. Identification of proteins with global effects on MMEJ:NHEJ balance.

To assess the global effect of proteins on MMEJ:NHEJ balance (**Fig. 1B & Fig. 2**), we computed for each KO the mean $\Delta\log_2$MMEJ:NHEJ over all 19 IPRs. Next, to identify proteins that significantly *favor MMEJ* or *favor NHEJ* independently of the chromatin state, we tested whether the mean $\Delta\log_2$MMEJ:NHEJ was different than zero by a Student's t-test followed by Benjamini-Hochberg multiple-testing correction of p-values. We called proteins to globally *favor MMEJ* (mean $\Delta\log_2$MMEJ:NHEJ < 0) or globally *favor NHEJ* (mean $\Delta\log_2$MMEJ:NHEJ > 0) with an estimated false-discovery rate (FDR) < 0.001.

5. *Identification of proteins with CCD: three-step linear modelling*

a. *Initial selection of proteins with any effect on MMEJ:NHEJ balance.*

To filter for proteins with any effect on MMEJ:NHEJ balance, we calculated the Z-score $\log_2$MMEJ:NHEJ for each 19 IPR in 519 KO gRNA samples (total of 9861) using the 33 mock KO gRNA samples (total of 627) to empirically estimate null-distributions. First, we fitted a normal distribution based on the mock KO $\log_2$MMEJ:NHEJ scores (n = 33) for each IPR and replicate separately (example IPR in **Supplementary Figure 2A**). Next, we standardized $\log_2$MMEJ:NHEJ scores of each sample using the mean and standard deviation of the fitted distributions (Supplementary Equation 4) (example IPR in **Supplementary Figure 2B**). Finally, we combined the Z-scores of the three independent replicates by Stouffer's method (Supplementary Equation 5) (**Supplementary Figure 2C**). After this transformation, 24.5% KO - IPR combinations (n = 2420) had an absolute Z-score >1.96, compared to only a 4.3% of mock KO - IPR combinations (n = 27). We retained a KO if the absolute Z-score was >1.96 in at least 2 out of 19 IPRs. A total of 352 KOs passed this filter. Of the 33 mock KO samples four passed the same criteria, suggesting an empirical FDR of 12%. Note that further filters are applied below for additional stringency. From the 352 proteins that passed this filter, 296 *favor MMEJ*, 47 *favor NHEJ* and 9 had mixed effects, i.e they *favor MMEJ* in some IPRs and *favor NHEJ* in others.

$$Z_{\text{MMEJ:NHEJ}} = \frac{\log_2 \text{MMEJ:NHEJ} - \mu_{\text{N}_{\text{null}}}}{\sigma_{\text{N}_{\text{null}}}} \tag{4}$$

Where N_{null} is a normal distribution fitted with mock KO samples (n = 33).

$$Z\text{-}score = \frac{\sum_{i=1}^{n} Z_{\text{MMEJ:NHEJ}}}{\sqrt{n}} \tag{5}$$

Where n is the number of replicates.

b. *Principal component regression.*

Next, among the remaining 352 proteins, we identified proteins with significant chromatin context dependencies (CCDs) across the entire set of 25 chromatin features. Because of the strong covariation among most chromatin features, we did this by principal component (PC) regression. This consists of dimension reduction using standard principal component analysis, followed by linear regression on the main PCs (**Supplementary Figure 3A**). This approach provides substantial robustness and avoids identification of fortuitous correlations with single chromatin features.

The sources of all chromatin feature tracks are summarized in **Supplementary Data 3**. Each of these 25 tracks was z-normalized. Z-scores were calculated as the $\log_2$ fold-difference of the signal over control (matching controls as provided by the respective studies)

in 2kb bins centered around each IPR insertion site. These values were subsequently converted into Z-scores using the mean and standard deviation of the chromatin feature signal in the TRIP pools, as previously done [3].

We then first assessed the number of PCs needed to explain most of the variance in the chromatin data of the 19 IPRs. For this we used *pls* package (version 2.8-1) in *R*. We selected the first three PCs, which together account for 76% of the variance. Adding a fourth PC to the model would only increase the explained variance by 6% (**Supplementary Figure 3B**). Closer inspection of the first three PCs revealed that each PC explained biologically relevant differences in chromatin contexts: PC1 mainly explained the difference between euchromatin and heterochromatin, PC2 mainly explained differences between heterochromatin types (Triple heterochromatin vs. H3K27me3) and PC3 mainly explained differences between euchromatin types (Enhancer/promoters vs. transcription) together with replication timing (**Supplementary Figure 3C**). Then, for each of the 354 KO and 4 mock samples, we constructed a linear model based on three PCs to predict the $\Delta\log_2$MMEJ:NHEJ scores. To assess the accuracy of this fit, we computed the p-value of the correlation between predicted $\Delta\log_2$MMEJ:NHEJ scores and measured $\Delta\log_2$MMEJ:NHEJ scores for each of the samples. After Benjamini-Hochberg correction of these p-values for multiple testing, 89 protein KOs and 1 mock KO passed the significance threshold at FDR cutoff 0.05. In RPE-1 cells, we performed this step using 10 available chromatin feature tracks.

c. Linear modeling to identify individual protein – chromatin feature links.

Finally, to identify the individual chromatin features that contribute to the CCDs, we fitted linear correlations between $\Delta\log_2$MMEJ:NHEJ of 89 proteins with significant CCDs and each of the 25 individual chromatin features (total of 2225). Based on this calculation, we identified individual protein – chromatin feature pairs with N-synergies, M-synergies or no synergies as follows:

A protein – chromatin feature pair is defined to have <u>N-synergy</u> when the protein *favors NHEJ* according to section *5a* in the data analysis workflow, and the linear fit has a positive slope (**Supplementary Figure 4A**). This positive slope implies that the ability of the protein to shift the balance towards NHEJ increases with increasing levels of the chromatin feature. It is also possible that a protein that *favors NHEJ* according to step *5a* shows a negative slope (**Supplementary Figure 4B**). However, such a negative correlation is likely to reflect an indirect effect. For example, IPRs with high H3K4me3 signals often exhibit low H3K9me3 signals and vice versa. Because the vast majority of molecular interactions in chromatin have so far been explained by the presence of a chromatin feature (e.g., a certain histone modification) rather than the absence of a chromatin feature, we focus on positive slopes for N-synergy and reject negative slopes as likely reflecting indirect correlations.

Conversely, we define a protein – chromatin feature pair as having <u>M-synergy</u> when the protein *favors MMEJ* (according to step *5a*) and the linear fit has a negative slope (**Supplementary Figure 4C**). Here, the negative slope implies that the ability of the protein to shift the balance towards MMEJ increases with increasing levels of the chromatin feature. Again, weaker effects with increasing levels of the chromatin feature (in this case a positive

slope; Supplementary Figure 4D) are most likely due to indirect correlations, and thus not considered to be M-synergy.

By these criteria, a few proteins showed both M- and N-synergy, with different chromatin features (Supplementary Figure 4E-F). We used the slope of the linear fits of M- or N- synergistic pairs as a measure of the synergy (synergy score). This score is set to 0 for protein – feature pairs without synergistic interactions as defined above. Of the 89 proteins with significant CCDs, 73 have M-synergies, 14 have N-synergies and 2 have mixed synergies.

Additionally, we fitted similar linear models for protein – chromatin feature combinations for the remaining 263 proteins that modulate the MMEJ:NHEJ balance (step *5a*) but did not pass the CCD significance threshold (step *5b*). We highlight some of these proteins in the main text, but always in connection with proteins with significant CCDs (Fig. 4B-D).

6. Estimation of chromatin context dependent MMEJ:NHEJ balance changes.

As stated above, the synergy score is the slope of the linear fit between Δlog_2MMEJ:NHEJ and a chromatin feature. The predicted effect size of a chromatin feature on the MMEJ:NHEJ balance (i.e., the dynamic range of Δlog_2MMEJ:NHEJ values across the entire genome, from the lowest to the highest level of the chromatin feature) not only depends on this slope, but also on the dynamic range of levels of this chromatin feature. To estimate this effect size, we first approximated this genome-wide dynamic range of each chromatin feature from the chromatin scores of 2,150 previously characterized randomly integrated IPRs [3] (grey distributions in Supplementary Figure 6A) as the difference between the bottom 0.5% and top 0.5% (Supplementary Figure 6A). We then multiplied this difference with the synergy score, resulting in a rough estimate of the genome-wide CCD Δlog_2MMEJ:NHEJ. For global and CCD Δlog_2MMEJ:NHEJ comparisons of each proteins (Supplementary Figure 6), we selected the maximum estimated CCD Δlog_2MMEJ:NHEJ of each protein. For this figure we classified proteins with CCDs into proteins with only CCDs effects ($FDR_{CCD} < 0.05$ & $FDR_{global} \geq 0.001$) and proteins with both CCDs and global effects ($FDR_{CCD} < 0.05$ & $FDR_{global} < 0.001$).

7. Estimation of screen KO penetrance

The effect sizes calculated above are likely to be underestimates, because the KO efficiencies after transfection of the gRNAs (Day 1) are expected to be less than 100%. Because we could not measure these efficiencies for all KOs directly (which would require gene-specific PCR for each KO), we obtained an approximate estimate as follows. We assumed that Day 1 transfections were equally efficient as the Day 6 transfections. From the latter, we calculated the mean editing efficiency (Supplementary Equation 1) of IPRs in transcriptionally active chromatin (n = 8). We focused on IPRs in transcriptionally active chromatin because they are more representative of the chromatin type that most gRNAs in the KO library target. We considered that a reporter is embedded in transcriptionally active chromatin when at least one of the transcription-related features TTseq, H3K36me3, POL2AS2 or POL2 had a chromatin Z-score higher than 0.5. Then, we calculated the average editing frequency of the mock transfected samples (n = 33) for each IPR and replicate (Supplementary Figure 10B). The

results suggest that the editing efficiency was in the range of 40-80%. This estimate is consistent with the efficiency of editing of the LBR gene with gRNA LBR2 after the first transfection, as measured on Day 5 (**Supplementary Figure 10A**), which was in the range 47.5%-70% for the 3 screen replicates. A similar control experiment was performed after the second transfection with consistent results (**Day 9, Supplementary Figure 10A**) However, we note that this estimation does not take into account the percentage of in-frame indels created by CRISPR/Cas9 or other gene editing products that do not lead to a protein KO.

8. Data visualization

We visualized CCDs of proteins as a heatmap (**Fig. 2D**) and as a Uniform Manifold Approximation and Projection (UMAP) plot (**Fig. 4C**). In the heatmap, we hierarchically clustered the synergy scores of every protein – chromatin feature pair using the "ward.D" algorithm in the pheatmap

package in R (version 1.0.12). The hierarchically clustered dendrogram (**Fig. 2D**) was divided intfour groups to highlight the main clusters observed in the heatmap. The UMAP was calculated withthe umap package (version 0.2.8.0) and two UMAP dimensions plotted as a scatterplot.

SUPPLEMENTARY TABLES

Supplementary Table 1: gRNA and primer sequences used in this manuscript.

Type	Name	Sequence
gRNA	LBR2	GCCGATGGTGAAGTGGTAAG
gRNA	POLQ_1	CGGACCCGGAGAGGAACTGG
gRNA	POLQ_2	TGCGTCGGAGTGGGAAACGG
gRNA	POLQ_3	AAGCTACTATTGGCAAACTG
gRNA	POLQ_4	TCTTTTTTACACCAAAACTG
DNA Primer	TAC0017	GTAGCCTTTCTGGCCCTAAAAT
DNA Primer	TAC0018	AAATGGCTGTCTTTCCCAGTAA
DNA Primer	TAC0007.1-24	ACACTCTTTCCCTACACGACGCTCTTCCGATCT**(N)10**GTCACAAGGGCCGGCCACA
DNA Primer	TAC0012	GTGACTGGAGTTCAGACGTGTGCTCTTCCGATCT
DNA Primer	TAC0009	AATGATACGGCGACCACCGAGATCTACACTCTTTCCCTACACGACGCTCTTCCGATCT
DNA Primer	TAC0159.1-96	CAAGCAGAAGACGGCATACGAGAT**(N)6**GTGACTGGAGTTCAGACGTGTGCTCTTCCGATCT

Supplementary Table 2: Overview of sequencing read numbers per sample in the screen

	Median			95% CI		
	R1	R2	R3	R1	R2	R3
Total (sample)	60408	53966	65220	[19366,96424]	[8611,194257]	[17047,171887]
Processed (sample)	49494	39226	46809	[15945,79317]	[6066,139428]	[12418,125270]
Processed (IPR)	2586	1920	2405	[850,4379]	[320,7700]	[560,7136]
Intact (IPR)	1436	728	969	[428,2697]	[103,3352]	[191,3251]
$NHEJ_{ins}$ (IPR)	516	563	725	[65,1278]	[48,2524]	[127,2468]
$MMEJ_{del}$ (IPR)	242	223	279	[39,2697]	[21,959]	[48,889]

SUPPLEMENTARY FIGURES

Supplementary Figure 1: Screen replicate reproducibility and distribution of Δ$\log_2$MMEJ:NHEJ values.

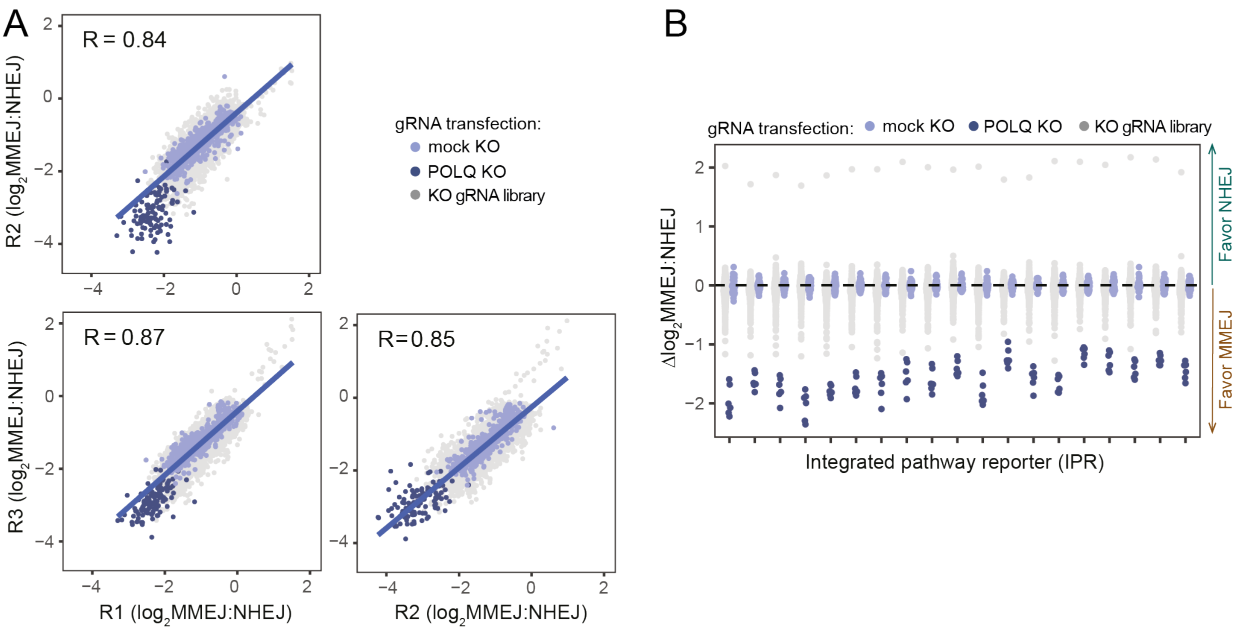

Supplementary Figure 1: Screen replicate reproducibility and distribution of Δ$\log_2$MMEJ:NHEJ values. A) Pairwise correlations of $\log_2$MMEJ:NHEJ values of individual IPRs between replicate experiments R1, R2 and R3, after application of quality filters as described in step 3 of the data processing. R denotes Pearson correlation coefficient. B) Dynamic range of Δ$\log_2$MMEJ:NHEJ balances after averaging of replicates (n = 3).

Supplementary Figure 2: z-transformation and combining of replicate measurements of log₂MMEJ:NHEJ values.

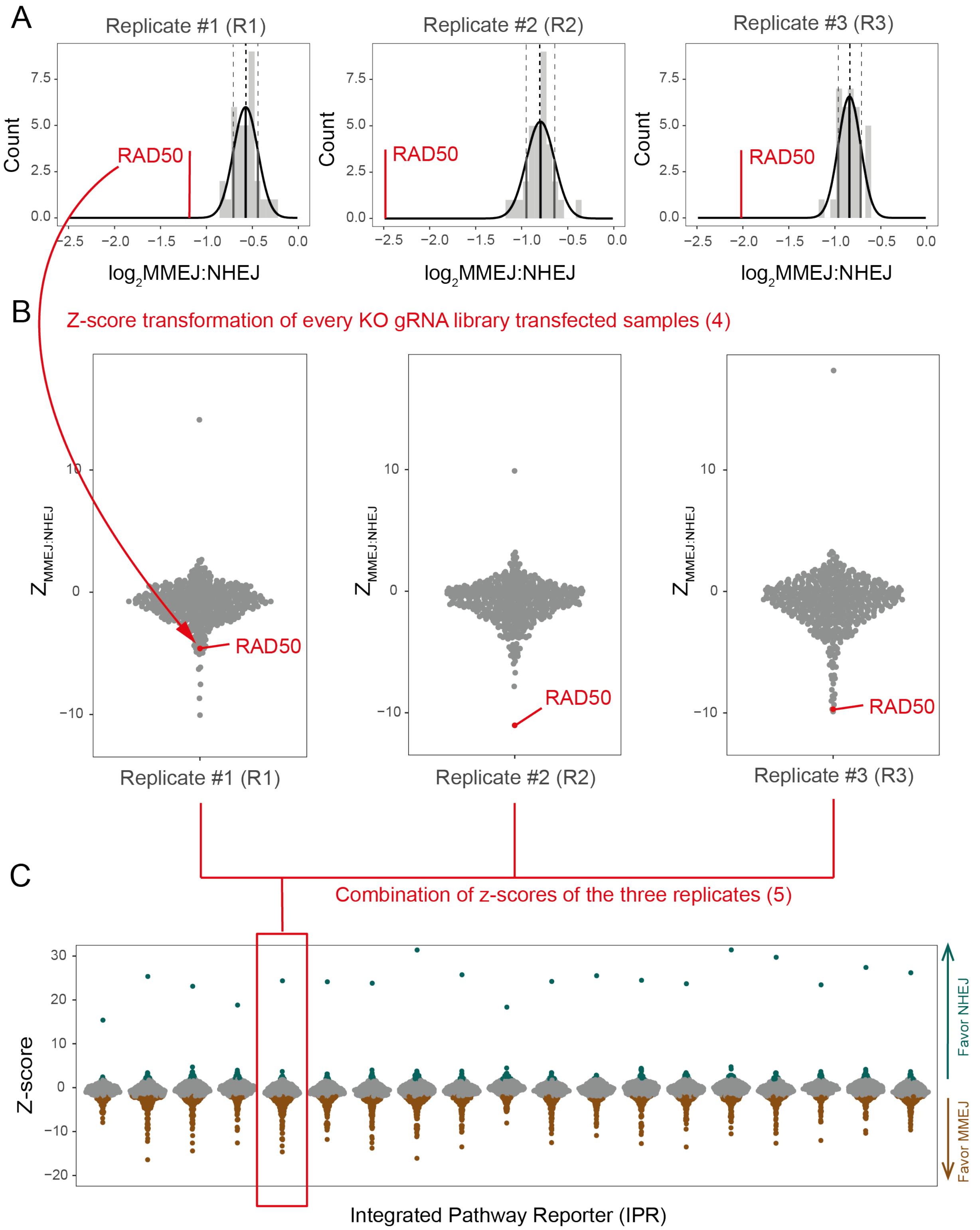

Supplementary Figure 2: z-transformation and combining of replicate measurements of log_2MMEJ:NHEJ values. A) Histogram of log_2MMEJ:NHEJ balance of mock KO transfected samples of a single IPR (IPR_barcode: CATTTCTGATCAATAA). The fitted normal distribution is depicted in black. Mean (black) and mean ± one standard deviation (grey) highlighted with vertical dotted lines. In red, log_2MMEJ:NHEJ balance of RAD50 KO is plotted as an example to illustrate the Z-score transformation for a single protein. A red arrow is displayed connecting RAD50 KO data point in

replicate #1 panel A and B. Each panel represents a different replicate and a similar arrow could be drawn for the other replicates as well. B) Beeswarm plot of the Z-score transformed log_2MMEJ:NHEJ balance of KO samples for a single reporter (CATTTCTGATCAATAA) (Supplementary Equation 4). C) Z-score transformed log_2MMEJ:NHEJ balance perturbations after combining three replicates for every MMEJ:NHEJ pathway reporters by the Stouffer's method (Supplementary Equation 5). A value outside the [-1.96,1.96] range is considered to be significant with a significance level of >95%. Positive values represent proteins that *favor NHEJ* (green dots and arrow) and negative values proteins that *favor MMEJ* (brown dots and arrow).

Supplementary Figure 3: Principal component regression analysis.

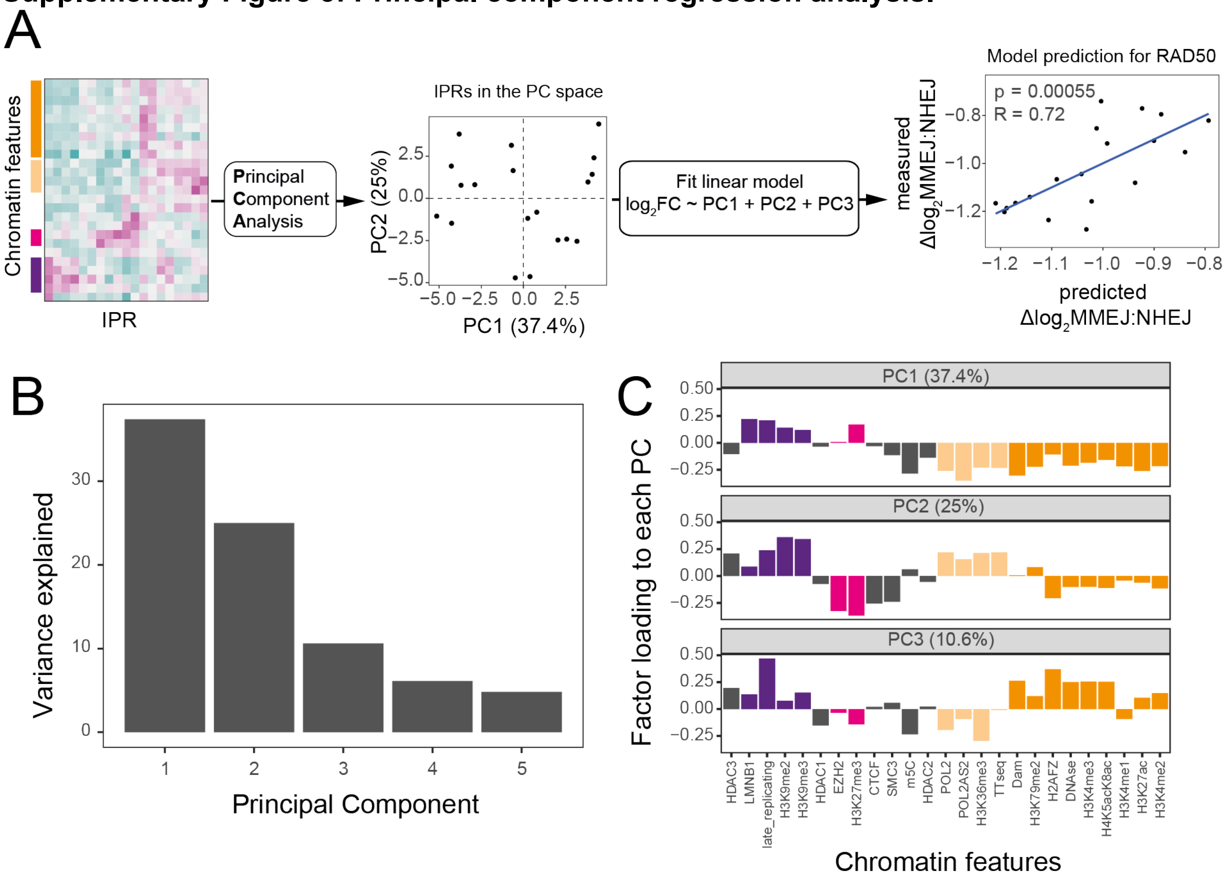

Supplementary Figure 3: Principal component regression analysis. A) Principal Component Regression workflow after averaging three replicates in the screen. First, a principal component analysis (PCA) was run with 25 chromatin feature values for each IPR. Second, exploration of the PCA revealed that the first three PCs recapitulate most of the chromatin feature variance. Third, a linear model with three principal components was ran for each protein and the performance was assessed by predicted vs. measured comparison. In the graph the Pearson's R and two-sided p-value is shown. B) Percentage of variance explained by the first five PCs. C) Bar graph showing the weight of each chromatin feature for PC1, PC2 and PC3. Bars are coloured according to the chromatin context they represent: Triple heterochromatin (purple), H3K27me3 heterochromatin (pink), transcription (light orange) and enhancers/promoters (orange).

Supplementary Figure 4: Examples of linear fit correlation with individual chromatin features.

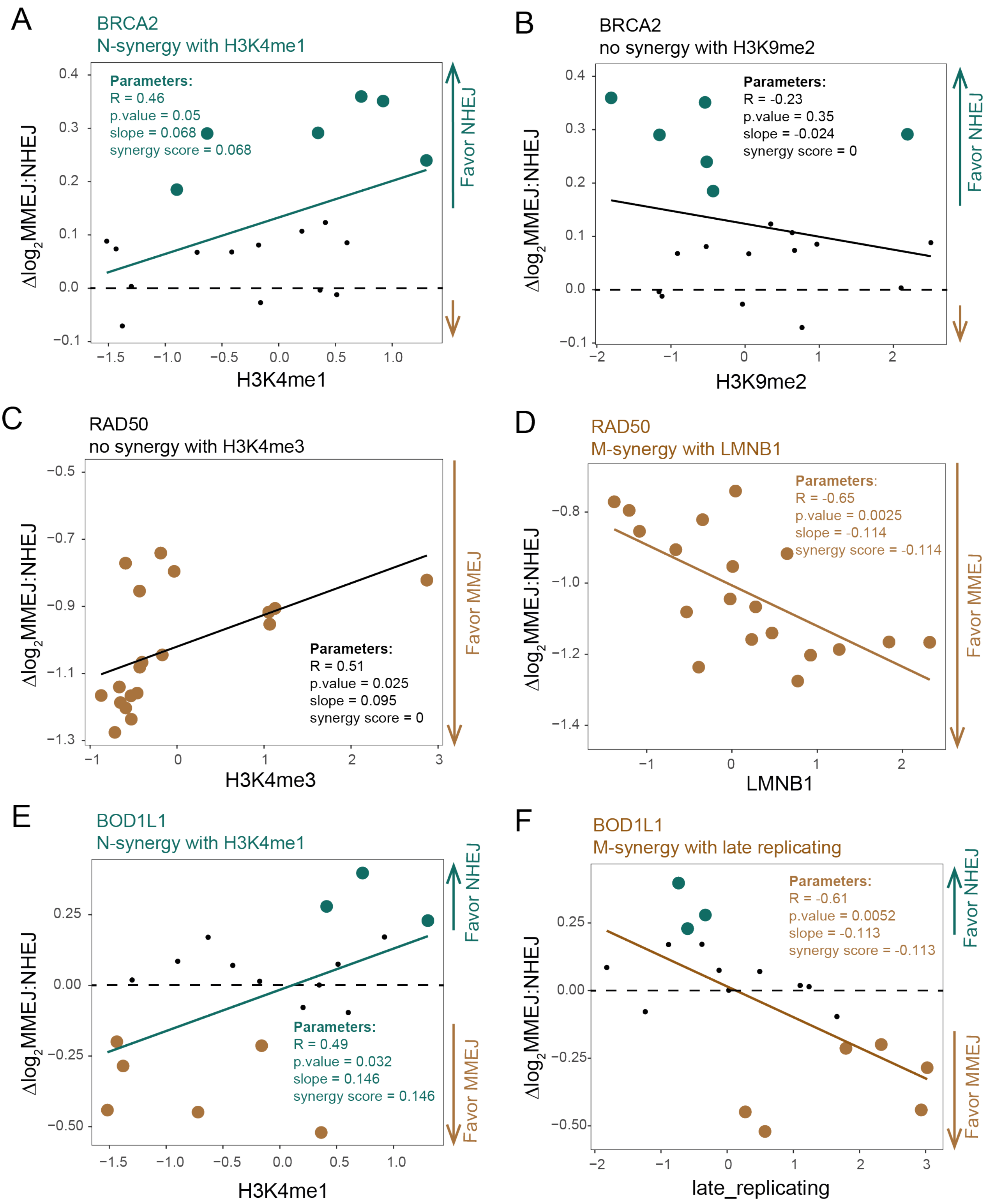

Supplementary Figure 4: Examples of linear fit correlation with individual chromatin features. Examples of three proteins with significant CCDs on individual chromatin features. All correlation plots show data of the 19 IPRs (n = 3), the linear regression fit, and regression analysis parameters that are relevant for the *synergy score*. (R = Pearson correlation coefficient, p.value = two-sided p value of the correlation coefficient, slope = slope of the linear fit, synergy score = final value of CCD interaction

between protein and chromatin feature after corrections). Color scheme of the figure shows if the protein - chromatin feature has an M-synergy (brown), N-synergy (green) or no synergy (black). A) N-synergy between BRCA2 and H3K4me1. B) No synergy between BRCA2 and H3K9me2. This interaction is explained by the absence of the chromatin feature and therefore is discarded (*favor NHEJ* and slope < 0). C) No synergy between RAD50 and H3K4me3. Same as for B applies here (*favor MMEJ* and slope > 0). D) M-synergy between RAD50 and interactions with the nuclear lamina (LMNB1). E) N-synergy between BOD1L and H3K4me1. F) M-synergy between BOD1L and late replicating chromatin.

Supplementary Figure 5: CCD pattern of DNA repair of the same Gene Ontology category

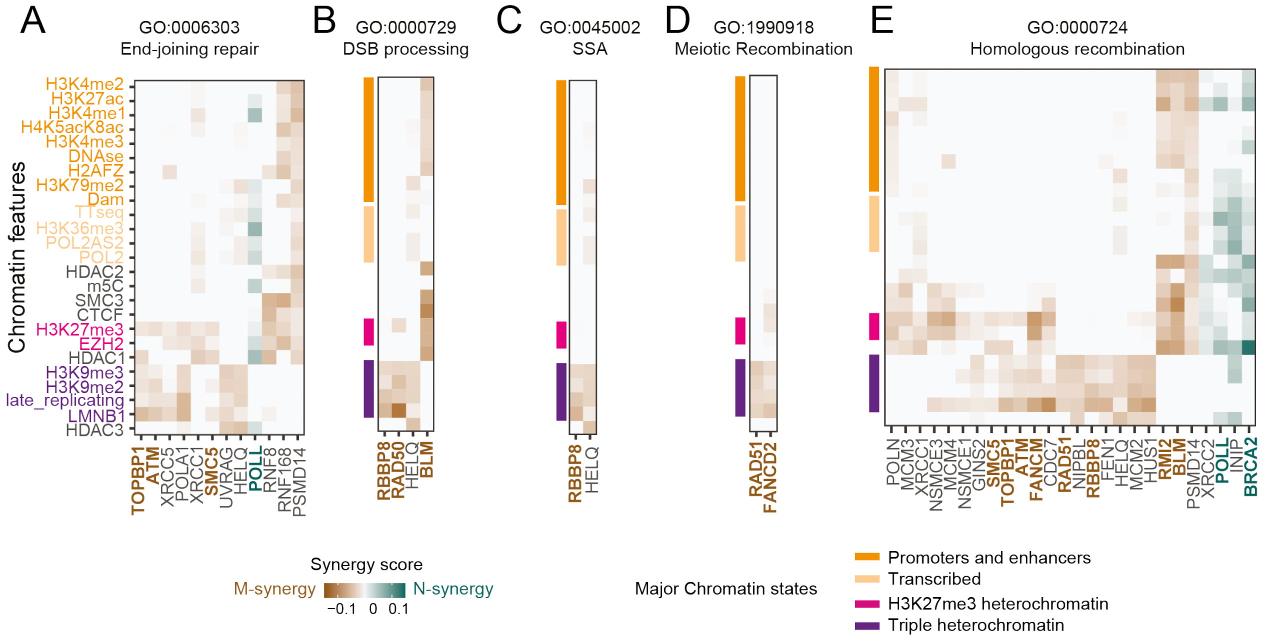

Supplementary Figure 5: M- and N-synergies of DNA repair proteins per Gene Ontology category. (**A-E**) CCD patterns of DNA repair proteins in (**A**) end-joining repair (GO:0006303) (**B**) DSB processing (GO:0000729), (**C**) single-strand annealing repair (GO:0045002), (**D**) meiotic recombination (GO:1990918), (**E**) homologous recombination (GO:0000724). Chromatin features are colored as in Fig. 1C and proteins arranged by CCD pattern similarity. Proteins highlighted in the text are highlighted in bold.

Supplementary Figure 6: Estimation of genome-wide dynamic ranges of chromatin features.

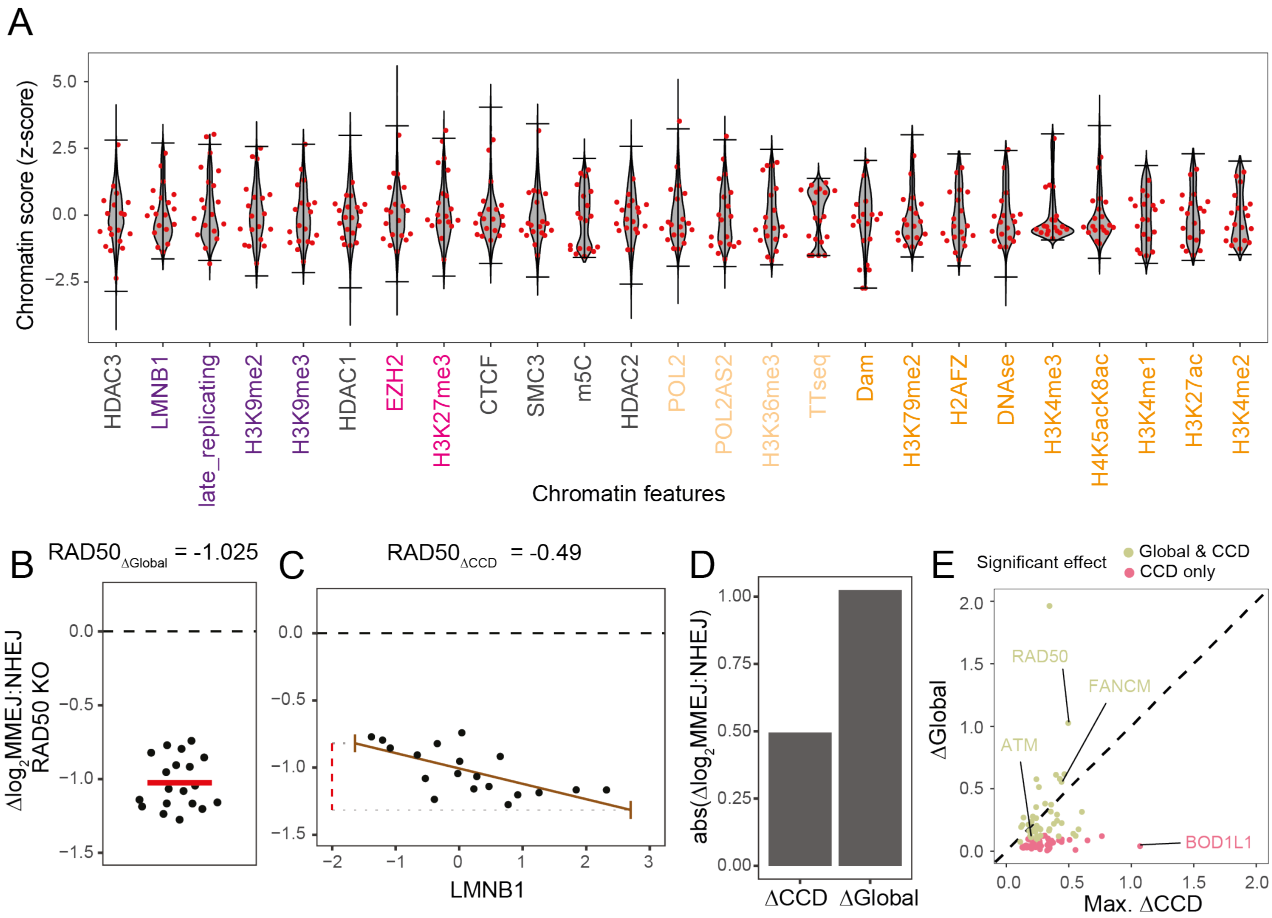

Supplementary Figure 6: Estimation of genome-wide dynamic ranges of chromatin features. A) In grey, distribution of genome-wide chromatin scores, with the two-sided 99% confidence interval (99CI) marked by the horizontal black lines (top 0.5% and bottom 0.5%). In red, chromatin scores of all 19 IPRs in clone 5 for which MMEJ:NHEJ balance was measured in the screen. **B**) Example of global Δlog_2MMEJ:NHEJ score of RAD50 KO. The global Δlog_2MMEJ:NHEJ score (ΔGlobal in short) is the mean Δlog_2MMEJ:NHEJ score (in red) of all 19 IPRs (individual datapoints, n = 3). **C**) Example on how the CCD Δlog_2MMEJ:NHEJ score (ΔCCD in short) is estimated. In this case, the linear fit of Δlog_2MMEJ:NHEJ scores with LMNB1 interaction levels (Same as in Fig. 2B). To estimate the genome-wide ΔCCD of RAD50, we extrapolated the linear fit to cover the two-sided 99CI of genome-wide LMNB1 levels ($LMNB1_{99CI}$ = [-1.64,2.7]) in brown. The maximum and minimum values of this extrapolated fit are taken as the Δlog_2MMEJ:NHEJ estimated in the absence and presence of LMNB1 interactions (0.005 and 0.995 percentiles). The differential between these two values is taken as the ΔCCD of RAD50. **D**) Direct comparison of CCD and Global Δlog_2MMEJ:NHEJ balance of RAD50. To compare between M- and N-synergies, which have different signs, we calculate the absolute CCD and Global Δog_2MMEJ:NHEJ balance. **E**) Scatter plot between ΔCCD and ΔGlobal scores for 89 proteins with CCDs. If a protein has larger global effect than CCD effect, it will be represented above the diagonal (dashed line). On the contrary, proteins with larger CCD effect will be below the diagonal. Overall, 89 proteins have similar CCD and global effects (they are next to the diagonal line).

Supplementary Figure 7: Effect size and knock-out penetrance estimation.

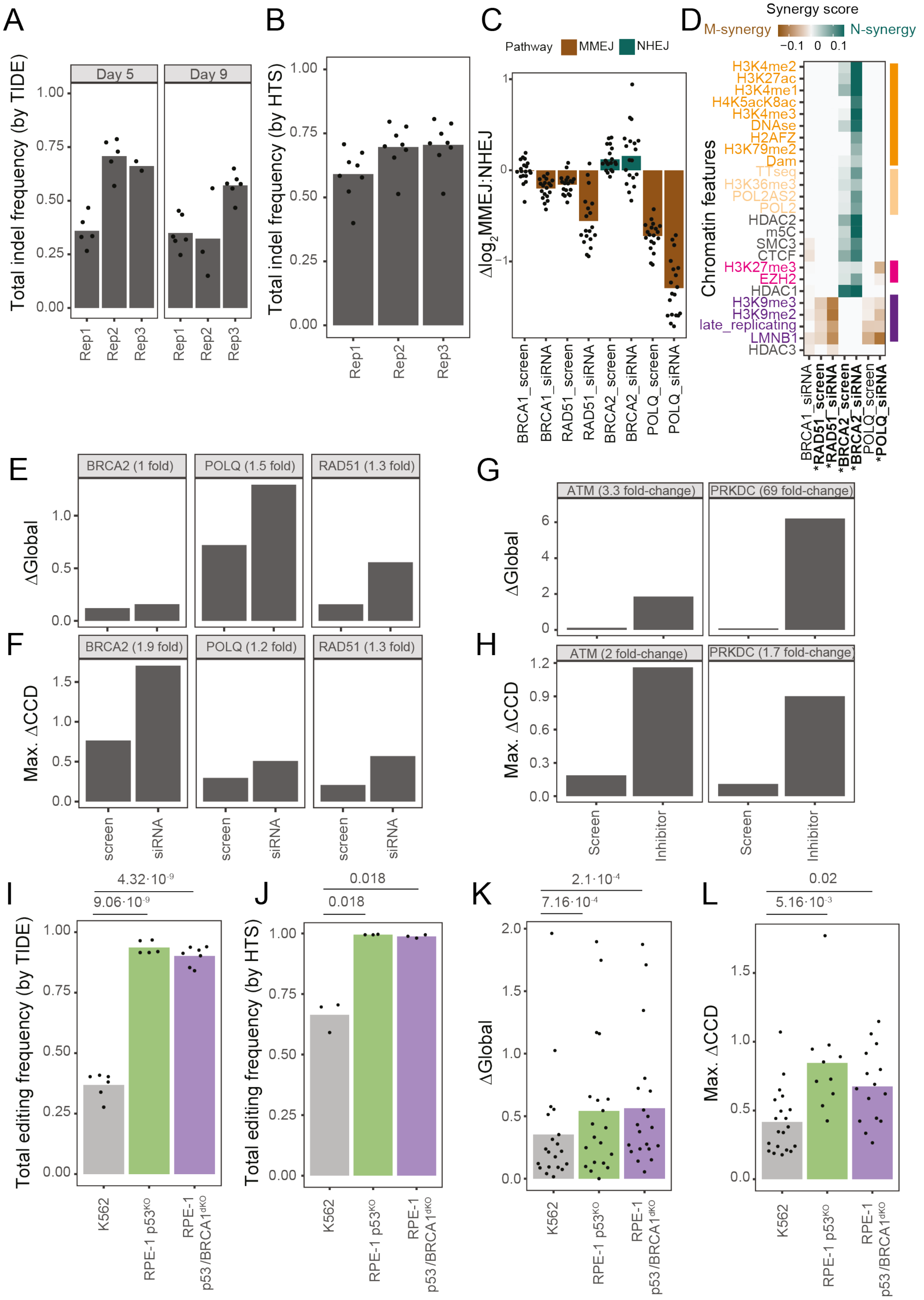

Supplementary Figure 7: Effect size and knock-out penetrance estimation. (**A**) Total indel frequency in control locus (endogenous *LBR* gene) measured by TIDE [2] in each replicate. Total indel frequency is calculated as the sum of the percentage of all detectable indels by the TIDE algorithm.

During the screening procedure, we measured editing frequency after the first transfection (Day 5) and the second transfection (Day 9) in every plate in the screen (n = 6). In this plot only TIDE data of sufficient quality (TIDE $R^2 > 0.75$) are included. Overall, TIDE estimated indel frequencies ranging from 0.37 to 0.71 for both transfections. (**B**) Complementary to TIDE, we calculated the mean total indel frequency in IPRs located in transcribed regions (n = 8) in K562 clone 5 for each replicate in mock transfected samples (n = 33). We chose IPRs in transcribed regions as they are most representative of the genes that we targeted in the KO screen. In this set-up, the total indel frequency is calculated as the frequency of reads with any insertion or deletion. By high-throughput sequencing, we estimated that the total editing frequency ranged from 0.59 to 0.7. Note that K562 cells are mostly triploid, and hence the percentage of cells with complete KO may be expected to be lower than these estimates. This is likely to account for the relatively small effect sizes observed in the K562 screen. To investigate the real extent of the effect-sizes, we performed the follow-up experiments in systems with higher penetrance. (**C-F**) First, we re-analyzed previously published siRNA knockdown data (n = 3). (**C**) Comparison of Δlog_2MMEJ:NHEJ scores of BRCA1, RAD51, BRCA2 and POLQ in the screen and after siRNA knock-downs from [3] (n = 19). In this experiment, the knock-down efficiency ranged from 50% to 80%. (**D**) CCD patterns of RAD51, BRCA2 and POLQ in the screen and siRNA experiment after averaging all three replicates. CCD patterns of BRCA1 only in the siRNA experiment. Chromatin features are colored as in **Fig. 1C**. (**E-F**) Knock-down of BRCA2, RAD51 and POLQ yield larger (**E**) global (ΔGlobal) and (**F**) CCD (Max. ΔCCD) log_2MMEJ:NHEJ scores than in the screen. We calculated ΔGlobal and ΔCCD as described in **Supplementary Figure 6**. (**G-H**) Second, we treated K562 clone 5 cells with small molecule inhibitors targeting ATM and PRKDC. Inhibitor treatments lead to (**G**) global (ΔGlobal) and (**H**) CCD (Max. ΔCCD) effect sizes larger than KO of the inhibited protein in the screen. We calculated ΔGlobal and ΔCCD as described in **Supplementary Figure 6**. Third, we tested the KO of 20 protein with CCDs in RPE-1 cells. (**I-J**) We measured the total indel frequency in RPE-1 cells compared to K562 (**I**) by TIDE (K562 n = 6, RPE-1 $p53^{KO}$ n = 5 and RPE-1 $p53/BRCA1^{dKO}$ n = 7) and (**J**) high-throughput sequencing (n = 3). With both techniques, we detected a nearly complete editing frequency in the *LBR* control locus in RPE-1 cells. Significance was assessed by two-sided Wilcoxon test with correction for multiple testing by the Benjamini-Hochberg method. (**K-L**) Δlog_2MMEJ:NHEJ in RPE-1 cells for the 20 targeted proteins, show higher (**K**) global (ΔGlobal) and (**L**) CCD (Max. ΔCCD) effect sizes than in K562 cells. We calculated ΔGlobal and ΔCCD as described in **Supplementary Figure 6**. Significance of the observed effects was measured by Wilcoxon test with correction for multiple testing by Benjamini-Hochberg method (n = 20). We conclude that the K562 screen underestimated the effect sizes of both the global and CCD effects, most likely due to low KO efficiencies.

Supplementary Figure 8: M- and N-synergies in RPE-1 cells.

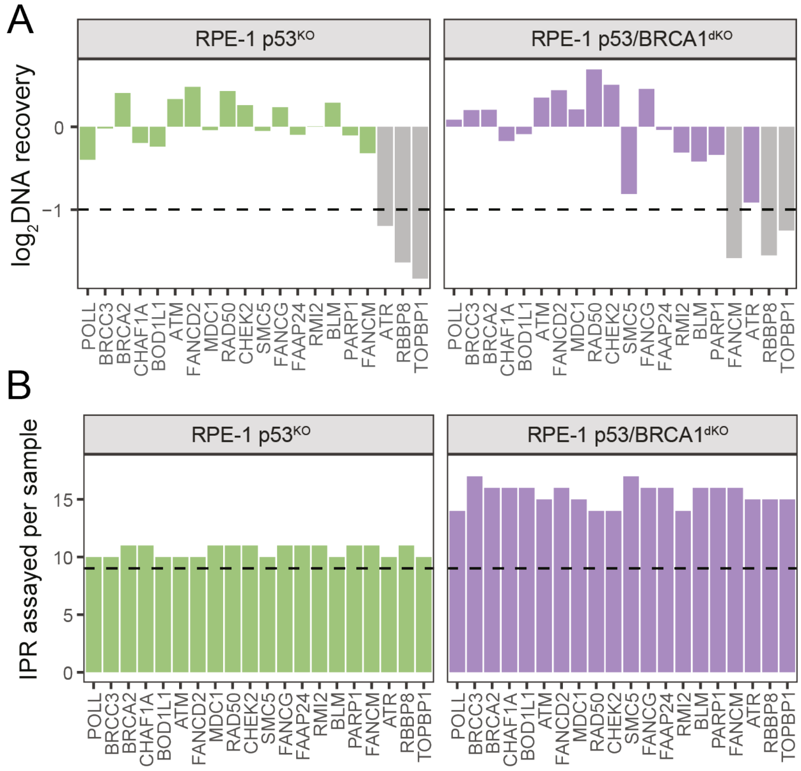

Supplementary Figure 8: M- and N- synergies in RPE-1 cells. A) log_2 DNA recovery ratio in KO samples compared to non-targeting control samples (NTC) for RPE-1 p53^{KO} and RPE-1 p53/BRCA1^{dKO} (n = 3). Samples with a 50% DNA content reduction compared to the NTC are colored in grey and marked in Fig. 3C with a X. These KO are likely to affect cell viability or proliferation in our experimental set-up, which translates into lower DNA recovery. **B**) Number of assayed IPRs in RPE-1 p53^{KO} and RPE-1 p53/BRCA1^{dKO}.

Supplementary Figure 9: ATM and DNAPK inhibitor effects.

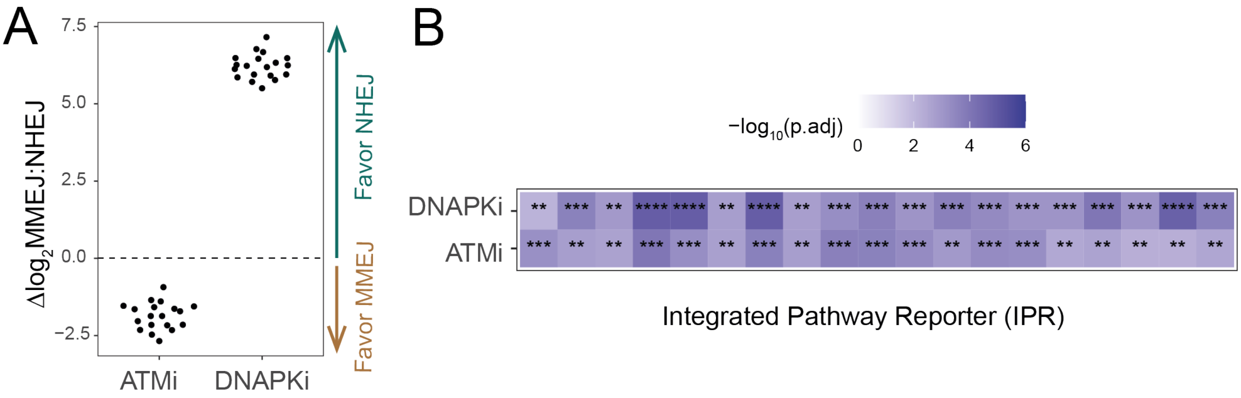

Supplementary Figure 9: ATM and DNAPK inhibitor effects. (**A**) Δlog_2MMEJ:NHEJ of ATM and DNAPKcs inhibitor of each IPR (n = 19). (**B**) Adjusted p-values of two-sided Student's t-test comparing Δlog_2MMEJ:NHEJ scores in ATM and DNAPKcs inhibited compared to the vehicle control (n = 3) for each reporter. This test was used to confirm significance of the changes in the log_2MMEJ:NHEJ.

Supplementary Figure 10: Impact of CCDs on human tumor genomes.

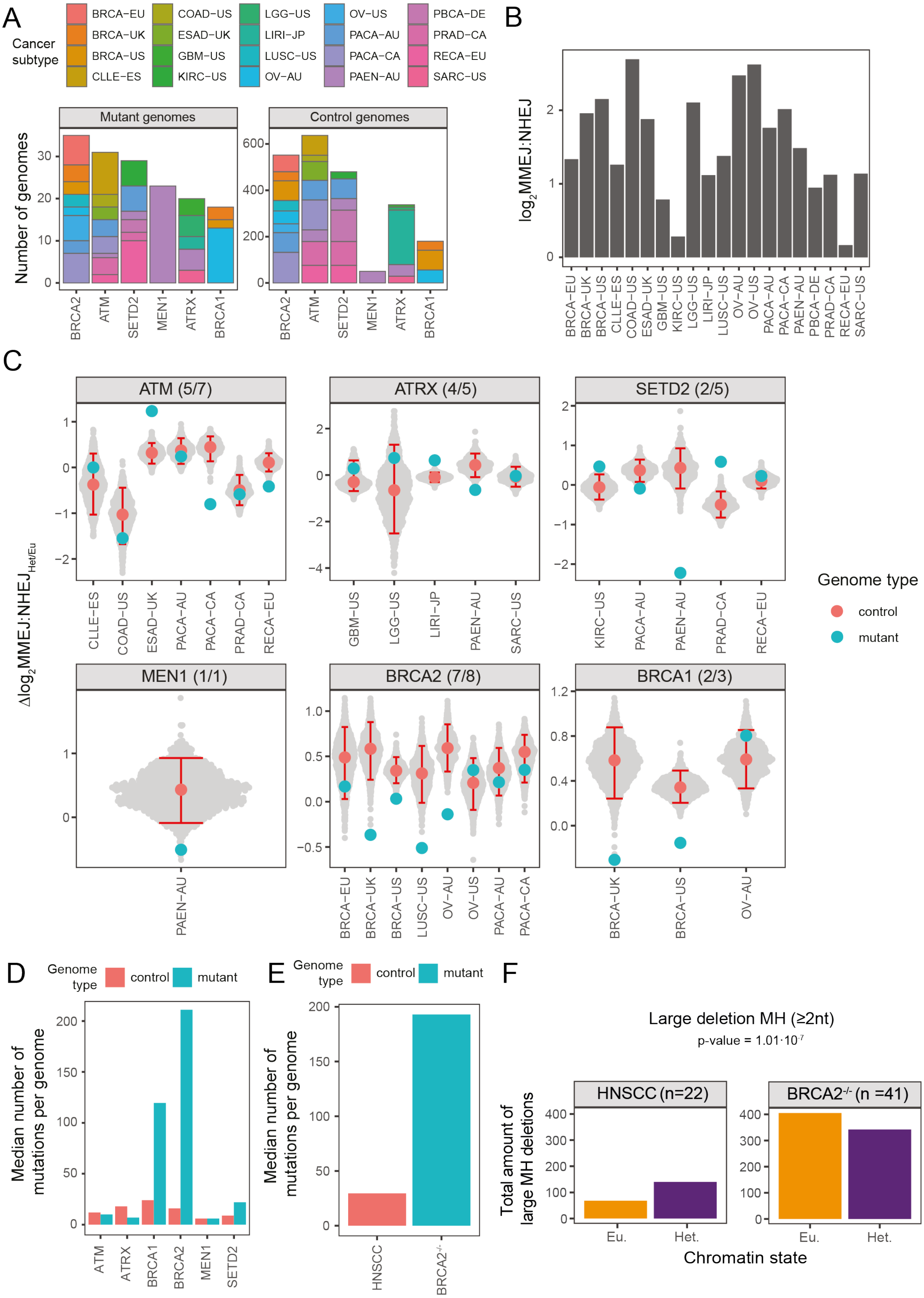

Supplementary Figure 10: Impact of CCDs on human tumor genomes. (**A**) Total number of human tumor genomes analyzed, classified by tumor types and driver genes in mutant cohorts (left) and controls (right). We analyzed genomes from 29 different tumor subtypes and 6 different driver genes.

(**B**) log_2MMEJ:NHEJ short deletion ratios per tumor subtype in control genomes. (**C**) Differential of log_2MMEJ:NHEJ balance ($\Delta log_2MMEJ:NHEJ_{Het/Eu}$) in heterochromatin (cLADs) and euchromatin (ciLADs) in controls and mutants, split by tumor type and driver gene. The null-distribution generated by bootstrapping of the control data is shown in grey for each tissue and driver cohort. Red dots indicate the mean $\Delta log_2MMEJ:NHEJ_{Het/Eu}$ of the control distribution (with red error-bars showing 95% CI); blue dots indicate mean $\Delta log_2MMEJ:NHEJ_{Het/Eu}$ of the indicated tumor type and driver gene. The fraction in the header of each panel summarizes the fraction of tumor types that follow the expect trend according to the K562 screen results. (**D-E**) Median number of MMEJ and NHEJ short deletions (**D**) in mutant cancer genomes compared to same tissue subtype controls by driver gene and (**E**) $BRCA2^{-/-}$ compared to genome-instable HPV_{neg} HNSCC. (**F**) Distribution of large deletions with MH at break sites counts over euchromatin (Eu.) and constitutive lamina-associated heterochromatin (Het.) in genome-instable HPV negative HNSCC and $BRCA2^{-/-}$ tumors. Two-sided p-value is shown in the figure.

SUPPLEMENTARY REFERENCES

1 Brinkman, E. K. *et al.* Kinetics and Fidelity of the Repair of Cas9-Induced Double-Strand DNA Breaks. *Mol Cell* **70**, 801-813 e806 (2018). https://doi.org:10.1016/j.molcel.2018.04.016

2 Brinkman, E. K., Chen, T., Amendola, M. & van Steensel, B. Easy quantitative assessment of genome editing by sequence trace decomposition. *Nucleic Acids Res* **42**, e168 (2014). https://doi.org:10.1093/nar/gku936

3 Schep, R. *et al.* Impact of chromatin context on Cas9-induced DNA double-strand break repair pathway balance. *Mol Cell* **81**, 2216-2230 e2210 (2021). https://doi.org:10.1016/j.molcel.2021.03.032

4 Schep, R., Leemans, C., Brinkman, E. K., van Schaik, T. & van Steensel, B. Protocol: A Multiplexed Reporter Assay to Study Effects of Chromatin Context on DNA Double-Strand Break Repair. *Front Genet* **12**, 785947 (2021). https://doi.org:10.3389/fgene.2021.785947

5 Yu, A. M. & McVey, M. Synthesis-dependent microhomology-mediated end joining accounts for multiple types of repair junctions. *Nucleic Acids Res* **38**, 5706-5717 (2010). https://doi.org:10.1093/nar/gkq379

6 Chan, S. H., Yu, A. M. & McVey, M. Dual roles for DNA polymerase theta in alternative end-joining repair of double-strand breaks in Drosophila. *PLoS Genet* **6**, e1001005 (2010). https://doi.org:10.1371/journal.pgen.1001005
