# Supplementary material for: Widespread chromatin context-dependencies of DNA double-strand break repair proteins
Source: Nat Commun. 2024 Jun 22;15:5334. doi: 10.1038/s41467-024-49232-x (PMC11193718; doi:10.1038/s41467-024-49232-x)
Supplement: Supplementary file 3 — Description of Additional Supplementary Files [file 41467_2024_49232_MOESM3_ESM.pdf]

Description of Additional Supplementary Files

File Name: Supplementary Data 1
Description: This file summarizes the information of the gRNAs used in this manuscript. Sheet 1 (Plate Specs) contains the information of the gRNAs used in the primary screen. Sheet 2 (Validation_gRNA_oligos) contains the full sequence of the gRNAs used in the validation experiments. The DNA sequence in this sheet contains the gRNA with the adapter sequences required to clone the gRNA in the destination vector.

File Name: Supplementary Data 2
Description: This file summarizes the publicly available chromatin datasets used to characterize the chromatin context of each IPR. It contains information such, cell line, chromatin feature, brief description, accession numbers and reference. Sheet 1 (ChIP_tracks) contains a list of all ChIP sequencing dataset used in the manuscript. Sheet 2 (others) contains a list of chromatin datasets that are not based on ChIP sequencing.

File Name: Supplementary Data 3
Description: This file includes the Δlog_2MMEJ:NHEJ of each IPR for all experiments performed in this manuscript and contains information such, cell line, perturbation, number of replicates, IPR assayed and the Δlog_2MMEJ:NHEJ. Sheet 1 (K562_screening_data) contains data of the primary screen. Sheet 2 (K562_inhibitor_data) contains data of the experiment with small molecule inhibitors in K562. Sheet 3 (RPE1_p53_KO) contains data of the knock-out experiment in RPE-1 $p53^{KO}$ cells. Sheet 4 (RPE1_p53_BRCA1_KO) contains data of the knock-out experiment in RPE-1 p53/$BRCA1^{dKO}$. Sheet 5 (RPE1_ATM_inhibitor) contains data of the experiment with small molecule inhibitors in RPE-1 cells.

File Name: Supplementary Data 4
Description: This file includes chromatin scores and genomic coordinates of each IPR measured in this manuscript. Sheet 1 (K562_clone5) contains data on K562 clone 5 IPRs (n = 19). Sheet 2 (RPE1_p53KO) contains data of the IPRs assayed in RPE-1 $p53^{KO}$ pool (n = 11). Sheet 3 (RPE1_p53_BRCA1_dKO) contains chromatin data of the IPRs assayed in RPE-1 p53/$BRCA1^{dKO}$ (n = 17).

File Name: Supplementary Data 5
Description: This file includes the main results of the manuscript and summarizes both global and CCD effects of DNA repair proteins. This sheet contains 6 sheets. Sheet 1 (Parameter_key) explains each column of the following sheets, where the data of each experiment is shown. Sheet 2 (K562_screen) contains data of the primary screen. Sheet 3 (K562_inhibitor) contains data of the experiment with small molecule inhibitors in K562. Sheet 4 (RPE1_p53_KO) contains data of the knock-out experiment in RPE-1 $p53^{KO}$ cells. Sheet 5 (RPE1_p53_BRCA1_dKO) contains data of the knock-out experiment in RPE-1 p53/$BRCA1^{dKO}$. Sheet 6 (RPE1_ATM_inhibitor) contains data of the small molecule inhibitors experiment in RPE-1 cells.

File Name: Supplementary Data 6
Description: This file includes all pairwise interactions, as described in BioGRID database, between proteins with CCDs used for the protein-protein interaction analysis and their cosine similarity score.

File Name: Supplementary Data 7
Description: This file includes mutation counts in human cancer genomes in LADs and iLADs. Sheet 1 (TCGA_data_indel) contains tumor data from TCGA database and includes information such as sample_ID, driver gene, TCGA project, whether sample belongs to the mutant or control cohort (sample) and MMEJ and NHEJ counts in LADs and iLADs. Sheet 2 (BRCA2-HNSCC_Webster_et_al_indel) contains similar mutation counts of $BRCA2^{+/+}$ and $BRCA2^{-/-}$ HNSCC tumors. Sheet 3 (BRCA2_HNSCC_Webster_et_al_SV) contains larger MMEJ deletion counts in $BRCA2^{+/+}$ and $BRCA2^{-/-}$ HNSCC tumors.
