# Supplementary material for: Widespread chromatin context-dependencies of DNA double-strand break repair proteins
Source: Nat Commun. 2024 Jun 22;15:5334. doi: 10.1038/s41467-024-49232-x (PMC11193718; doi:10.1038/s41467-024-49232-x)
Supplement: Supplementary file 11 — Reporting Summary [file 41467_2024_49232_MOESM11_ESM.pdf]

Corresponding author(s): Rene H. Medema and Bas van Steensel

Last updated by author(s): Apr 26, 2024

Reporting Summary

Nature Portfolio wishes to improve the reproducibility of the work that we publish. This form provides structure for consistency and transparency in reporting. For further information on Nature Portfolio policies, see our Editorial Policies and the Editorial Policy Checklist.

Statistics

For all statistical analyses, confirm that the following items are present in the figure legend, table legend, main text, or Methods section.

n/a	Confirmed	
☐	☒	The exact sample size (*n*) for each experimental group/condition, given as a discrete number and unit of measurement
☐	☒	A statement on whether measurements were taken from distinct samples or whether the same sample was measured repeatedly
☐	☒	The statistical test(s) used AND whether they are one- or two-sided<br>*Only common tests should be described solely by name; describe more complex techniques in the Methods section.*
☐	☒	A description of all covariates tested
☐	☒	A description of any assumptions or corrections, such as tests of normality and adjustment for multiple comparisons
☐	☒	A full description of the statistical parameters including central tendency (e.g. means) or other basic estimates (e.g. regression coefficient) AND variation (e.g. standard deviation) or associated estimates of uncertainty (e.g. confidence intervals)
☐	☒	For null hypothesis testing, the test statistic (e.g. *F*, *t*, *r*) with confidence intervals, effect sizes, degrees of freedom and *P* value noted<br>*Give P values as exact values whenever suitable.*
☒	☐	For Bayesian analysis, information on the choice of priors and Markov chain Monte Carlo settings
☒	☐	For hierarchical and complex designs, identification of the appropriate level for tests and full reporting of outcomes
☐	☒	Estimates of effect sizes (e.g. Cohen's *d*, Pearson's *r*), indicating how they were calculated

Our web collection on statistics for biologists contains articles on many of the points above.

Software and code

Policy information about availability of computer code

Data collection	Protein-protein interaction database: BioGRID (release version 4.4.209)
Data analysis	The code generated for this manuscript is deposited in: https://github.com/vansteensellab/CCD_repair_protein_project<br><br>Previously published software is described below:<br>Custom Indel and IPR scoring pipeline: (https://github.com/vansteensellab/DSB_TRIP_protocol)<br>IndelClassification (https://github.com/ferrannadeu/indelsClassification)<br>BRASS (https://github.com/cancerit/BRASS)<br><br>R packages used in the manuscript:<br>pls R package (version 2.8-1) = Used for principal component regression<br>lsa R package (version 0.73.3) = Calculate cosine similarity<br>igraph R package (version 1.3.4) = Compute interaction cliques<br>pheatmap R package (version 1.0.12) = Data visualization<br>umap package (version 0.2.8.0) = Data visualization

For manuscripts utilizing custom algorithms or software that are central to the research but not yet described in published literature, software must be made available to editors and reviewers. We strongly encourage code deposition in a community repository (e.g. GitHub). See the Nature Portfolio guidelines for submitting code & software for further information.

Data

Policy information about availability of data

All manuscripts must include a data availability statement. This statement should provide the following information, where applicable:

- Accession codes, unique identifiers, or web links for publicly available datasets
- A description of any restrictions on data availability
- For clinical datasets or third party data, please ensure that the statement adheres to our policy

All data generated for this manuscript have been deposited in the Sequence Read Archive (BioProject no. PRJNA882344).
In this manuscript, other publicly available datasets have been used as well, and corresponding data can be found in:
1- Data used to profile chromatin-context of Integrated pathway reporter in K562 and RPE-1 is publicly available (see table S5 for accession codes).
2- DNA repair protein siRNA knock-down (presented in figures Fig. S7C-F) raw data are deposited in the Sequence Read Archive (PRJNA686952) and processed data in github (https://osf.io/cywxd/)
3- Sporadic human tumor data is available from TCGA (https://portal.gdc.cancer.gov/projects), PCAWG (https://dcc.icgc.org/pcawg) and the full list of driver mutations identified by the pan-cancer analysis of whole genomes (PCAWG) consortium was obtained for all available cancer subtypes (https://dcc.icgc.org/releases/PCAWG/driver_mutations). The datasets and tumors identifiers used in for this manuscript are listed in Table Supplementary 9.
4- Unique identifiers for all chromatin maps used in this manuscript can are listed on Supplementary Data 3.

Research involving human participants, their data, or biological material

Policy information about studies with human participants or human data. See also policy information about sex, gender (identity/presentation), and sexual orientation and race, ethnicity and racism.

Reporting on sex and gender	Not applicable
Reporting on race, ethnicity, or other socially relevant groupings	Not applicable
Population characteristics	Not applicable
Recruitment	Not applicable
Ethics oversight	Not applicable

Note that full information on the approval of the study protocol must also be provided in the manuscript.

Field-specific reporting

Please select the one below that is the best fit for your research. If you are not sure, read the appropriate sections before making your selection.

☒ Life sciences ☐ Behavioural & social sciences ☐ Ecological, evolutionary & environmental sciences

For a reference copy of the document with all sections, see nature.com/documents/nr-reporting-summary-flat.pdf

Life sciences study design

All studies must disclose on these points even when the disclosure is negative.

Sample size	Three replicates of screen and inhibitor experiments were analyzed, following the convention in the field.<br>Three replicates of the RPE1 and K562 experiments were analyzed, following the convention in the field.<br>Protein-protein interaction analysis was performed with every pair available in the BioGRID database as described in the Methods section "Comparison to protein-protein interaction data".<br>We used human tumor sequencing data and further analyze these for chromatin dependent mutation accumulation as described in "Chromatin context dependent pathway activity in tumors" section in Methods and sample size shown in Figure Supplementary 10A.
Data exclusions	Replicates of the CRISPR screen were discarded based on low editing efficiency as described in "Screening replicates" subsection in the supplementary Methods section. The semi-automated pipetting procedure sometimes leads to low editing frequencies and/or no editing. To detect such events and discard these replicates, we incorporated in our experiments technical controls (editing controls) to assess the efficiency of the editing procedure at day 5 and end-point of the screen.<br>Human tumor data were discarded if any they fulfill any of these criteria: (i) exhibit markers of DNA repair deficiencies, (ii) displayed mutational signatures indicative of previous mutagenic treatments and (iii) have less than 500 substitutions genome-wide. Any of these criteria would act as a confounding factor of our read-out.
Replication	Reproducibility of the data was measured as reproducibility of the MMEJ:NHEJ measurements. As described in the sample size section, every experiment was performed at least three times following the convention in the field.<br>We validated the CCD of human tumor data by analyzing different tumor types when possible. As reported in Figure Supplementary 10C, we observe that results are generally conserved across different tumor types.

Randomization	The position in the arrayed screen was performed based on the order in the screening library. Downstream processing allocation (lysis, sequencing index selection, PCR and high-throughput sequencing) was random or performed per replicate (sequencing lanes). Index selection of inhibitor experiments and data in RPE-1 cells was random.
Blinding	Blinding was not relevant in this study. All the data generated for this manuscript were analyzed in a automated manner.

Reporting for specific materials, systems and methods

We require information from authors about some types of materials, experimental systems and methods used in many studies. Here, indicate whether each material, system or method listed is relevant to your study. If you are not sure if a list item applies to your research, read the appropriate section before selecting a response.

Materials & experimental systems

n/a	Involved in the study	
☒	☐	Antibodies
☐	☒	Eukaryotic cell lines
☒	☐	Palaeontology and archaeology
☒	☐	Animals and other organisms
☒	☐	Clinical data
☒	☐	Dual use research of concern
☒	☐	Plants

Methods

n/a	Involved in the study	
☒	☐	ChIP-seq
☒	☐	Flow cytometry
☒	☐	MRI-based neuroimaging

Eukaryotic cell lines

Policy information about cell lines and Sex and Gender in Research

Cell line source(s)	Genetically modified K562 cell line. This cell line was originally acquired from the ATCC and subsequent genetic modifications were performed as described in "R. Schep et al., Impact of chromatin context on Cas9-induced DNA double-strand break repair pathway balance. Mol Cell 81, 2216-2230 e2210 (2021)."<br>RPE-1 p53KO and RPE-1 p53/BRCA1dKO in this manuscript were generated by "Zimmermann, M. et al. CRISPR screens identify genomic ribonucleotides as a source of PARP-trapping lesions. Nature 559, 285-289 (2018)." and were kindly shared by the Jonkers' lab at the Netherlands Cancer Institute.
Authentication	None of the cell lines were authenticated after the genetically modifying it.
Mycoplasma contamination	Cell lines were tested negative regularly for Mycoplasma contamination.
Commonly misidentified lines<br>(See ICLAC register)	None of the cell lines used in this study were listed in the ICLAC register (version 12)

Plants

Seed stocks	Not applicable
Novel plant genotypes	Not applicable
Authentication	Not applicable
